# 2351. Concordance Between Sources of COVID-19 Vaccination Information Among Children Aged 5–18 years, May 2021–March 2023

**DOI:** 10.1093/ofid/ofad500.1972

**Published:** 2023-11-27

**Authors:** Sarah Hamid, Laura D Zambrano, Regina Simeone, Margaret M Newhams, Natasha B Halasa, Katherine E Fleming-Dutra, Amber Orzel, Michael J Wu, Adrienne G Randolph, Angela P Campbell

**Affiliations:** Epidemic Intelligence Service, Centers for Disease Control and Prevention; Coronavirus and other Respiratory Viruses Division, National Center for Immunization and Respiratory Disease, CDC, Atlanta, Georgia; Centers for Disease Control and Prevention, Atlanta, GA; Centers for Disease Control and Prevention, Atlanta, GA; Boston Children's Hospital, Boston, Massachusetts; Vanderbilt University Medical Center, Nashville, Tennessee; Centers for Disease Control and Prevention, Atlanta, GA; Boston Children's Hospital, Boston, Massachusetts; Centers for Disease Control and Prevention, Atlanta, GA; Boston Children's Hospital, Harvard Medical School, Boston, Massachusetts; Centers for Disease Control and Prevention, Atlanta, GA

## Abstract

**Background:**

Documentation of COVID-19 vaccination is crucial for monitoring vaccine uptake and effectiveness. U.S. providers are required to document all COVID-19 vaccines administered in jurisdictional Immunization Information Systems (IIS). The end of the Public Health Emergency in May 2023 and subsequent commercialization of COVID-19 vaccines could reduce reporting to IIS. Parent report is an alternative source of vaccination data but could result in vaccination status misclassification through recall bias, social desirability bias, or other mechanisms.

**Methods:**

We compared numbers of parent-reported and documented COVID-19 vaccine doses received by patients aged 5–18 years admitted to 31 Overcoming COVID-19 Network hospitals in 23 states. Documentation comprised vaccine cards, electronic medical records, pediatrician records, or IIS. We calculated percent agreement and a kappa statistic for concordance. We used logistic regression to estimate associations between vaccination status agreement and demographic and clinical characteristics.

**Results:**

Among 2,296 children hospitalized during 5/16/2021–3/20/2023, agreement between the number of parent-reported and documented doses was 89.5% (95% CI: 88.2–90.7%), with a kappa of 0.79 (95% CI: 0.77–0.82) (Table 1, Table 2). Most discordant pairs (223/242) were due to parents reporting more doses than were found in documented sources. Testing positive for SARS-CoV-2 was associated with 1.6 higher odds of agreement compared with testing negative (Table 3). Compared with children of white race, the odds of agreement for children of Asian race and multiple races were 0.41 and 0.61 lower, respectively. The median time between hospital admission and parent interview was greater for children with discordant vaccine status (54 days [IQR: 20–106]) than for those with concordant status (14 days [IQR: 3–49]).

**Table 1.**
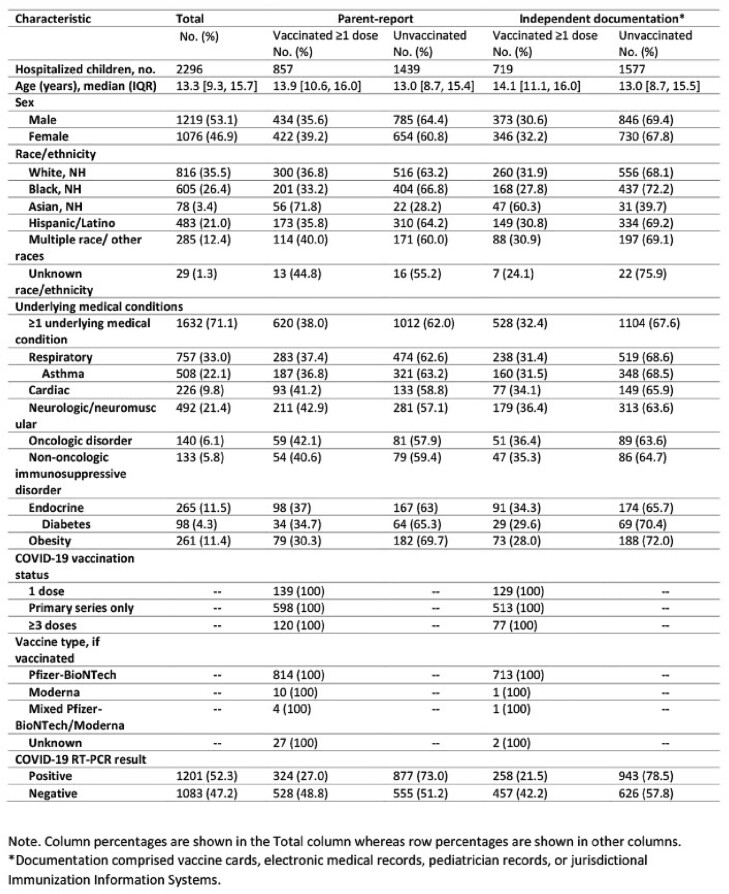
Characteristics of hospitalized children aged 5–18 years in concordance analysis of parent-reported versus documented COVID-19 vaccination status, May 2021 – March 2023, n=2,296

**Table. 2.**
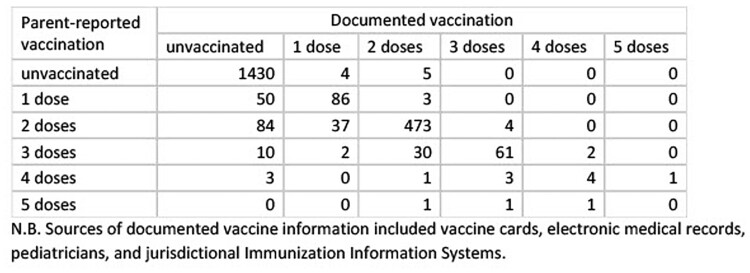
Comparison of parent-reported versus documented COVID-19 vaccination among hospitalized children aged 5–18 years (n=2,296), May 2021 – March 2023

**Table 3.**
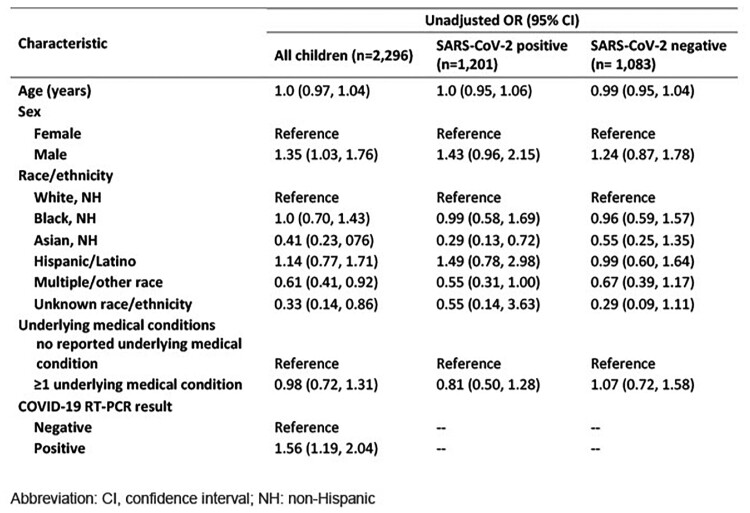
Unadjusted odds ratios (ORs) for agreement of total doses between parent-reported and documented COVID-19 vaccination status by demographic and clinical characteristics (n=2,296), May 2021-March 2023

**Conclusion:**

Parent-reported COVID-19 vaccination status agrees substantially with documented status among pediatric patients. Reducing the time from hospital admission to parent interview could improve parent report accuracy. Differences in the accuracy of parent report by demographic groups and SARS-CoV-2 test result should be considered in COVID-19 vaccine effectiveness studies.

**Disclosures:**

**Regina Simeone, PhD**, Pfizer: Stocks/Bonds **Natasha B. Halasa, MD, MPH**, Merck: Grant/Research Support|Quidell: Grant/Research Support|Quidell: donation of kits|Sanofi: Grant/Research Support|Sanofi: vaccine support

